# Tranexamic acid for significant traumatic brain injury (The CRASH-3 trial): Statistical analysis plan for an international, randomised, double-blind, placebo-controlled trial

**DOI:** 10.12688/wellcomeopenres.14700.2

**Published:** 2018-09-26

**Authors:** Ian Roberts, Antonio Belli, Amy Brenner, Rizwana Chaudhri, Bukola Fawole, Tim Harris, Rashid Jooma, Abda Mahmood, Temitayo Shokunbi, Haleema Shakur, CRASH-3 trial collaborators

**Affiliations:** 1 Clinical Trials Unit, LSHTM, London, WC1E 7HT, UK; 2 The National Institute for Health Research Surgical Reconstruction and Microbiology Research Centre, Queen Elizabeth Hospital, Birmingham, UK; 3 RMC-LSHTM Research Collaboration Centre (RLRCC), Holy Family Hospital, Rawalpindi, Pakistan; 4 COMUI-LSHTM Research Collaboration Centre (CLRCC), University College Hospital, Ibadan, Nigeria; 5 Barts Health and Queen Mary University, London, UK; 6 Aga Khan University Hospital, Karachi, Pakistan

**Keywords:** Antifibrinolytic, clinical trial, intracranial bleeding, tranexamic acid, traumatic brain injury

## Abstract

**Background: **Worldwide, traumatic brain injury (TBI) kills or hospitalises over 10 million people each year. Early intracranial bleeding is common after TBI, increasing the risk of death and disability. Tranexamic acid reduces blood loss in surgery and death due to bleeding in trauma patients with extra-cranial injury. Early administration of tranexamic acid in TBI patients might limit intracranial bleeding, reducing death and disability. The CRASH-3 trial aims to provide evidence on the effect of tranexamic acid on death and disability in TBI patients. We will randomly allocate about 13,000 TBI patients (approximately 10,000 within 3 hours of injury) to an intravenous infusion of tranexamic acid or matching placebo in addition to usual care. This paper presents a protocol update (version 2.1) and statistical analysis plan for the CRASH-3 trial.

**Results: **The primary outcome is head injury death in hospital within 28 days of injury for patients treated within 3 hours of injury (deaths in patients treated after 3 hours will also be reported). Because there are reasons to expect that tranexamic acid will be most effective in patients treated immediately after injury and less effective with increasing delay, the effect in patients treated within one hour of injury is of particular interest. Secondary outcomes are all-cause and cause-specific mortality, vascular occlusive events, disability based on the Disability Rating Scale and measures suggested by patient representatives, seizures, neurosurgical intervention, neurosurgical blood loss, days in intensive care and adverse events. Sub-group analyses will examine the effect of tranexamic acid on head injury death stratified by time to treatment, severity of TBI and baseline risk.

**Conclusion:** The CRASH-3 trial will provide reliable evidence of the effectiveness and safety of tranexamic acid in patients with acute TBI.

**Registration: **International Standard Randomised Controlled Trials registry ( ISRCTN15088122) 19/07/2011, and ClinicalTrials.gov ( NCT01402882) 25/07/2011.

## List of abbreviations

AEs: Adverse events; CI: Confidence interval; CONSORT: Consolidated Standards of Reporting Trials; CRASH-2: Clinical randomisation of an anti-fibrinolytic in significant haemorrhage; CRASH-3: Clinical randomisation of an anti-fibrinolytic in significant head injury; CT: Computed tomography; DMP: Data Management Plan; DIC: Disseminated intravascular coagulation; GCS: Glasgow coma scale; HIV: Human immunodeficiency virus; ICH-GCP: International Conference on Harmonization – Good Clinical Practice; IBMS: Intracranial Bleeding Mechanistic Sub-study; ICU: Intensive Care Unit; LSHTM: London School of Hygiene and Tropical Medicine; PAI-1: Plasminogen activator inhibitor-1; RR: Relative risk; SAEs: Serious adverse events; SUSARs: Suspected unexpected serious adverse reaction; TB: Tuberculosis; TBI: Traumatic brain injury; TPA: Tissue plasminogen activator; TXA: Tranexamic acid; UK: United Kingdom; USA: United States of America

## Introduction

There are more deaths each year from injuries than from HIV, TB and malaria combined ^1^. Worldwide, over ten million people are killed or hospitalised each year after traumatic brain injury (TBI) ^2^. Road traffic crashes are the main cause of serious TBI. Most of the deaths are in low and middle income countries where the TBI incidence is high due to rapidly increasing motorisation ^3^. Africa and South Asia have particularly high TBI rates, due to their high rate of traffic crashes ^3^.

Early intracranial bleeding is common after TBI and increases the risk of death and disability ^4^. In patients with moderate and severe TBI, intracranial bleeding may continue after hospital admission and for several hours after injury ^5– 7^. Abnormal coagulation with increased fibrinolysis may worsen intracranial bleeding ^8^. High levels of fibrin degradation products are often seen in the first three hours ^9^. These patients have a higher risk of intracranial bleeding and death.

Tranexamic acid reduces bleeding by inhibiting the enzymatic breakdown of fibrinogen and fibrin. A systematic review of randomized trials of tranexamic acid in surgery showed that tranexamic acid reduces blood loss by one third ^10^. Tranexamic acid also reduces mortality in bleeding trauma patients. In 2011, the CRASH-2 trial showed that administration of tranexamic acid to poly-trauma patients within three hours of injury reduces deaths due to bleeding by about one third ^11, 12^. In 2017, the WOMAN trial showed that administration of tranexamic acid to women with post-partum haemorrhage within three hours of delivery reduces deaths due to bleeding by about one third ^13^. Later in 2017, results from an individual patient-level data meta-analysis of randomised trials of tranexamic acid in acute severe bleeding showed that whilst immediate treatment substantially improves survival, the survival benefit decreases by around 10% for every 15 min of treatment delay until 3 h, after which there was no benefit ^14^.

The CRASH-3 trial is an international, randomized, placebo-controlled trial to quantify the effects of early administration of tranexamic acid on death and disability in patients with TBI. We expect tranexamic acid to be more effective than placebo in reducing death and disability in patients with TBI. We published the protocol before the start of the trial ^15^. In September 2016 we increased the sample size from 10,000 to 13,000 patients. In this paper, we provide the reason for this increase. This statistical analysis plan was completed and submitted for publication before the treatment allocation was un-blinded.

## Trial methods

### Trial design and patients

The CRASH-3 trial is an international, multi-centre, randomised, parallel group, double blind, placebo controlled trial of the effects of early administration (within 3 hours of injury) of tranexamic acid on death and disability in TBI patients. Adults with TBI within 3 hours of injury, with any intracranial bleeding on CT scan or who have a GCS of 12 or less, and no significant extra cranial bleeding are potentially eligible. The time window for eligibility was originally within 8 hours of injury but in 2016 the protocol was amended to limit recruitment to patients who are within 3 hours of injury. We will randomly allocate approximately 13,000 TBI patients who meet the eligibility criteria to receive tranexamic acid or placebo. The primary outcome is head injury death in hospital within 28 days of injury in patients treated within 3 hours of injury.

### Trial registration

The CRASH-3 trial was prospectively registered at the International Standard Randomised Controlled Trials registry ( ISRCTN15088122) on 19 July 2011, and ClinicalTrials.gov on 25 July 2011 ( NCT01402882).

### Ethics approval

The Medical Research and Ethics Committee and Health Research Authority reviewed the protocol and supporting documents for the CRASH-3 trial and provided a favourable ethical opinion on 19 July 2012 (Research Ethics Committee Reference 12/EE/0274). The Medicines and Healthcare Products Regulatory Agency authorised the CRASH-3 trial on 8 August 2012 (Reference 17072/0007/001-0001). Favourable ethical opinion was received from the Observational/Interventions Research Ethics Committee at the London School of Hygiene and Tropical Medicine (LSHTM) on 17 November 2011 (Reference 6060). The Medical Research and Ethics Committee and Health Research Authority reviewed the protocol and supporting documents for the IBMS and provided a favourable ethical opinion on 8 June 2016 (Research Ethics Committee Reference 12/EE/0274). Favourable ethical opinion was received from the Observational/Interventions Research Ethics Committee at the London School of Hygiene and Tropical Medicine on 24 May 2016 (Reference 11535). Important protocol modifications to the CRASH-3 trial will be submitted to and reviewed by the Medicines and Healthcare Products Regulatory Agency, Medical Research and Ethics Committee and Health Research Authority, and registries updated as appropriate. All ethical approvals have been reviewed in support of publication of the CRASH-3 trial protocols ^15, 16^.

### Consent to participate

TBI patients are physically and mentally incapable of providing informed consent to participate in a clinical trial. As acknowledged in the Declaration of Helsinki, patients who are incapable of giving consent are an exception to the general rule of informed consent in clinical trials (34). In the CRASH-3 trial, patients are unable to provide consent and so consent is sought from the patient’s relative, legal representative or the responsible clinician. If and when the patient regains capacity to provide informed consent, they are informed about the trial and written consent sought to continue their participation in the trial. If a patient or patient representative declines consent, they are withdrawn from the trial. For patients who were included in the trial but did not regain capacity, written informed consent is sought from a relative or legal representative. The requirements of relevant local and national ethics committees are adhered to at all times. The CRASH-3 trial includes consent to extract data from patient medical records. Collecting CT scan data for the IBMS is consistent with the consent procedure used in the CRASH-3 trial. It would be impractical to re-consent patients or relatives/legal representatives to access CT scans, particularly for patients who have deceased or are disabled as a result of their injuries where re-consent would be distressing and unwelcome. LSHTM and national Ethics Committees extended their approvals to extract CT data from the CRASH-3 trial without further patient consent. Patients who withdrew from the main CRASH-3 trial would not be included in the IBMS.

### Randomisation and masking

An independent statistical consultant from Sealed Envelope Ltd (UK) prepared and secured the randomisation codes. They were then given to the drug packers so that treatment packs could be prepared in accordance with the randomisation list. We will randomise TBI patients eligible for inclusion to receive active treatment (tranexamic acid) or placebo (sodium chloride, 0.9%) intravenously. Half of the patients will receive tranexamic acid whilst the other half will receive placebo. Baseline information will be collected on the entry form and the next lowest consecutively numbered pack will be taken from a box of eight treatment packs. If the treatment ampoule is confirmed as intact, the patient is considered randomised. Entry form data should be sent to the Trial Coordinating Centre as soon as possible. Both participants and study staff (site investigators and trial coordinating centre staff) will be masked to allocation. An emergency un-blinding service is available. The tranexamic acid (Cyklokapron® Injection) used in the trial is manufactured by Pfizer Ltd Sandwich (UK). The South Devon Healthcare NHS Trust (UK) manufacture the matching placebo (sodium chloride, 0.9%). Ampoules and packaging are identical in appearance. The blinding is done by Brecon Pharmaceuticals Limited (Hereford, UK). The blinding process involves removal of the original manufacturer’s label and replacement with the clinical trial label bearing the randomisation number (the pack identification). All pack label texts are identical for both tranexamic and placebo. We check the coding of blinded ampoules by randomly testing each batch of trial treatments and doing high performance liquid chromatography to check the contents.

### Trial procedures

When eligibility has been confirmed and the consent procedure followed, each patient is assigned a uniquely numbered treatment pack and are thus randomly allocated to receive tranexamic acid (loading dose 1g over 10 minutes followed by an infusion of 1g over 8 hours) or matching placebo. Once randomised, we will collect patient outcome data even if the trial treatment is not given. Outcome data are collected four weeks (28 days) after randomisation, at discharge from the randomising hospital or at death (whichever occurs first).

### Sample size

We originally estimated that a trial with about 10,000 patients would have 90% power (two-sided alpha of 1%) to detect a 15% relative reduction (20% to 17%) in mortality. However, whilst the trial was underway, new research suggested a shorter therapeutic window than 8 hours ^12, 13^. The new data showed that tranexamic acid is most likely to improve outcome if given soon after injury and would be unlikely to improve outcome if given beyond three hours of injury. In response to this evidence, in September 2016, we changed the primary outcome to head injury death in hospital within 28 days of injury in patients randomised within 3 hours of injury. We also limited recruitment to within 3 hours. We increased the sample size to 13,000 to get enough patients (about 10,000 as per the original sample size calculation) within 3 hours of injury to confirm or refute an early benefit. Statisticians at the London School of Hygiene and Tropical Medicine (University of London) have reviewed the sample size calculations.

## Statistical analysis plan

### Trial profile

We will show the flow of study participants in a Consolidated Standards of Reporting Trials (CONSORT) diagram. This will include the total number of participants randomised into the trial divided by treatment arm. Each treatment arm will detail the number of patients who withdrew consent, the number of patients for whom baseline data was collected, the number lost to follow up and the number of patients for whom outcome data was collected. Because TBI is a life-threatening emergency, we do not ask clinicians to complete a screening log since this would take time away from important clinical work. We will report the number of patients included in the primary and secondary analyses, the reasons for any post-randomisation exclusions and the number lost to follow-up. We will count patients that did not fulfil the eligibility criteria or did not receive their allocated treatment as having deviated from the protocol. Their data will be included in the intention to treat analysis. If a patient or their representative withdraws consent for data collection, we will use only data up to the point of withdrawal in the analysis.

### Baseline characteristics

The trial Entry Form ( Supplementary File 1) will be used to collect baseline information including age, sex, time since injury, systolic blood pressure, Glasgow coma score, pupil reaction, and if relevant, the location of intracranial haemorrhage. To check that randomisation produced similar groups, we will describe the baseline characteristics of each group with frequencies, percentages, means, medians and standard deviations as appropriate.

### Primary outcome

The primary outcome is head injury death in hospital within 28 days of injury *among patients randomised within 3 hours of injury.* Because there are strong scientific reasons to expect that tranexamic acid will be most effective in patients treated within an hour of injury and less effective with increasing treatment delay, the effect in patients treated within one hour of injury is of particular interest. Cause of death is assessed and recorded by the responsible clinician. Although some misclassification is inevitable, all-cause mortality combines causes of death that might be affected by tranexamic acid (e.g. head injury death) with causes that we do not expect to be affected by tranexamic acid (e.g. sepsis death) and this will dilute any effect.

In the original trial protocol, the primary end-point included all patients randomised within 8 hours of injury. This was based on the CRASH-2 trial results which showed that giving tranexamic acid to patients with traumatic extra-cranial bleeding within eight hours of injury reduces death due to bleeding and all-cause mortality ^11^. However, pre-specified subgroup analyses showed that the effect of tranexamic acid depends on the time interval between the injury and start of treatment ^12^. Treatment within three hours of injury substantially reduced death due to bleeding and all-cause mortality whereas treatment started after three hours appeared to increase death due to bleeding and had no effect on all-cause mortality. Although we expected early treatment to be most effective, we did not expect such a qualitative time to treatment interaction. It is unusual for a treatment to be beneficial in one subgroup and completely ineffective or harmful in another.

In response to the CRASH-2 trial results, research was conducted into the mechanism of action of tranexamic acid in trauma patients. This research provided a biological explanation for the time to treatment interaction. Early fibrinolysis is common after trauma and is associated with increased mortality ^17– 19^. Trauma triggers the early release of tissue plasminogen activator (TPA), the enzyme that converts plasminogen into the fibrinolytic enzyme plasmin ^20, 21^. Plasmin activation leads to fibrinolysis and profuse bleeding. TPA levels peak about 30 minutes after injury and plasmin levels peak around one hour ^21^. By inhibiting early fibrinolysis, tranexamic acid prevents early exsanguination ^17^. However, the effects are short lived. About two hours after injury, plasminogen activator inhibitor-1 (PAI-1) levels start to increase reaching a peak at three hours ^21^. PAI-1 inhibits fibrinolysis ^22^. This might explain why early tranexamic acid treatment is so important ^12^. The adverse effects of late tranexamic acid administration could be due to PAI-1-induced suppression of fibrinolysis and the onset of thrombotic disseminated intravascular coagulation (DIC). By inhibiting fibrinolysis, tranexamic acid might worsen DIC. Although the pathology is thrombotic, due to consumption of clotting factors, thrombotic DIC usually manifests as bleeding. Because TBI patients show similar coagulation changes, a similar time to treatment interaction is possible ^23– 25^.

In 2016 we obtained results from the WOMAN trial of the effect of tranexamic acid on death due to bleeding after post-partum haemorrhage (the results were published in 2017). The WOMAN trial provided more evidence in support of a time to treatment interaction. Treatment within three hours significantly reduced death due to bleeding but there was no evidence of any benefit beyond three hours ^13^. In light of the accumulating evidence that tranexamic acid treatment is unlikely to be effective when started more than three hours after injury, in September 2016, we restricted CRASH-3 trial recruitment to patients within 3 hours of injury and changed the primary end point accordingly.

In 2017, we published an individual patient-level data meta-analysis of randomised trials of tranexamic acid in acute severe bleeding. The results showed that immediate treatment improved survival by more than 70% (OR 1·72, 95% CI 1·42–2·10; p<0·0001) but thereafter, the survival benefit decreased by about 10% for every 15 min of treatment delay until 3 hours, after which there was no benefit ^14^. It is reasonable to expect a similar decline in treatment benefit in the context of acute intracranial bleeding. Because the treatment effect in patients treated within 3 hours of injury will be a weighted average of the effects in the first, second and third hours after injury, and most patients (about 80%) are recruited in the second and third hours, the overall effect could be diluted towards the null. For this reason the effect of treatment in patients treated within an hour of injury is of particular scientific interest. It is important to bear in mind that if tranexamic acid is shown to be safe and effective, time to treatment in clinical practice can be much shorter than in a clinical trial since there is no requirement for consent procedures and the treatment can be given earlier in the clinical pathway (e.g. pre-hospital).

In summary, although intracranial bleeding can continue up to 24 hours after injury, research published since the start of the CRASH-3 trial showed that treatment started beyond 3 hours of injury is unlikely to be effective and that even within the first three hours, earlier treatment is more likely to be of benefit. For these reasons, we changed the primary outcome to head injury death in patients randomised within 3 hours of injury with a focus on the effect in the first hour. All outcomes for patients treated after three hours of injury will be presented separately.

### Primary analysis

The main analyses will compare those allocated tranexamic acid versus those allocated placebo, on an intention to treat basis (irrespective of whether they received the allocated treatment). The primary analyses will be presented as relative risks and 95% confidence intervals. Kaplan-Meier estimates for the time to each of the primary and secondary outcomes will also be plotted (with their associated log-rank p-values).

### Sensitivity analysis

TBI patients who have a GCS of 3 and bilateral un-reactive pupils have a very poor prognosis, with a mortality risk of about 75%. The inclusion in the CRASH-3 trial of such severely injured patients, who may have little potential to benefit from the trial treatment, would bias the treatment effect towards the null. We will therefore conduct a sensitivity analysis on the primary endpoint that excludes patients with a GCS 3 and bilateral unreactive pupils.

### Missing data

Given the progress of data collection so far, we expect that loss to follow-up will be minimal (i.e. less than 1% missing data on the primary outcome) and so we will not impute missing values.

### Sub-group analyses

We will define all subgroups according to variables measured before randomisation. We will carry out the following subgroup analyses for head injury deaths.

**(a) Time to treatment**

**Figure 1.**
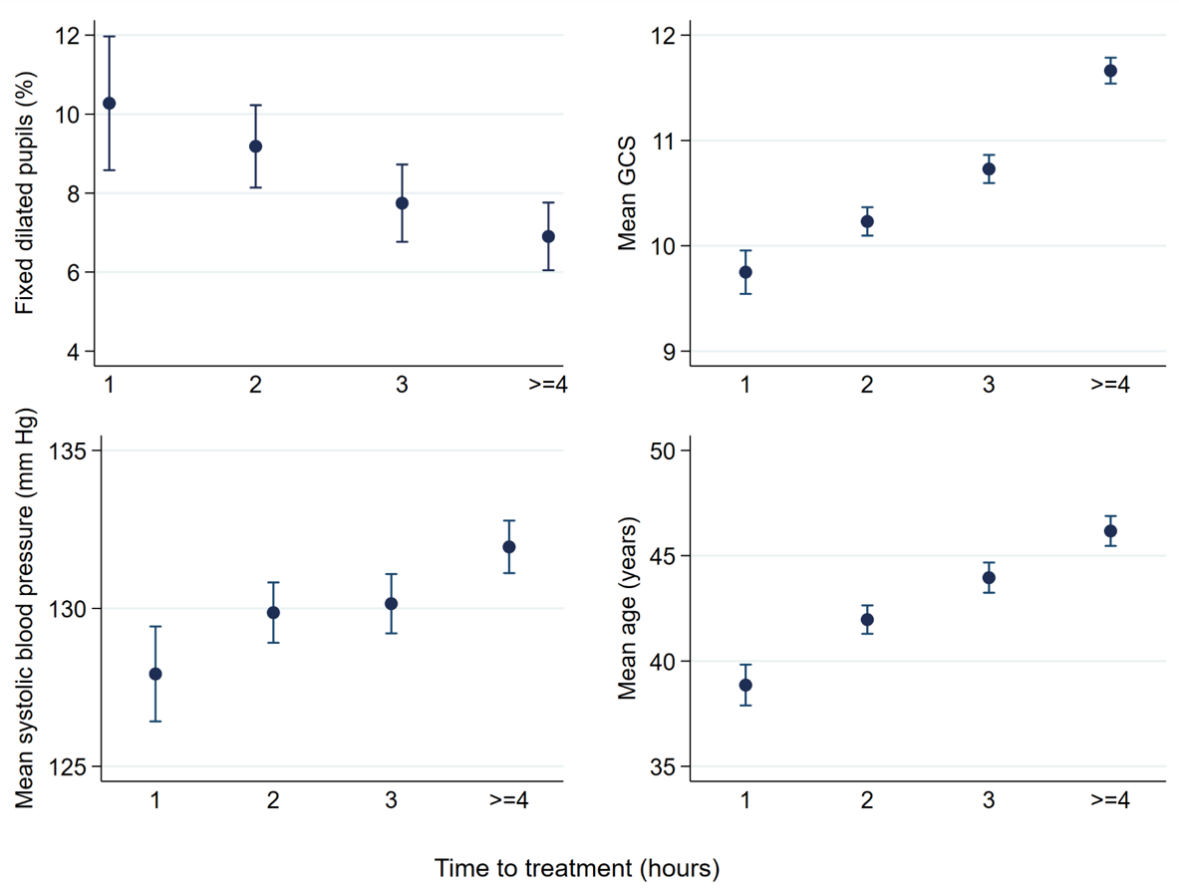
Relationship between baseline prognostic variables (dilated pupils, GCS, blood pressure and age) and time to treatment in hours. Based on blinded data on approximately 10,000 patients from the CRASH-3 trial.

We expect that the effect of tranexamic acid on death from head injury will vary by time to treatment with earlier treatment being most effective. We will examine this hypothesis by conducting sub-group analysis of the effect of tranexamic acid according to the estimated time interval between injury and randomisation (≤1, >1–≤3, >3h). Because TBI severity (GCS and pupil response), SBP and age could confound the impact of time to treatment on treatment effectiveness ( Figure 1), we will control for these variables in a multivariable model. Because there is prior evidence to expect a time to treatment interaction, we do not require as strong evidence against the null hypothesis of homogeneity as we might usually require. Most trials lack power to detect heterogeneity in treatment effects and the lack of a statistically significant interaction does not mean that the overall treatment effect applies to all TBI patients. Because there is prior evidence that late treatment may be ineffective or harmful, we will consider the results of the time to treatment sub-group analysis in the context of the existing trial data (including data from the CRASH-2 trial) on the time to treatment interaction with tranexamic acid and rely more on scientific judgment than on statistical tests. Because missing data for time to treatment is minimal, only patients with time to treatment data will be included in the analyses.

**(b) Severity of head injury**

We will examine the effect of tranexamic acid on death from head injury stratified by the severity of TBI at baseline. We will examine three sub-groups: mild (GCS 13-15), moderate (GCS 9-12) and severe (GCS 3-8). We will use interaction tests to see whether the effect of the treatment (if any) differs across these subgroups. We will also assess the impact of baseline severity on the treatment effect in a regression analysis that includes continuous terms for severity and its square (because of potential non-linearity of the treatment effect). Because time to treatment, SBP and age could confound impact of severity on treatment effectiveness, we will control for these variables. We do not expect the effect of tranexamic acid to vary substantially by severity of TBI and unless there is strong evidence against the null hypothesis of homogeneity of effects (i.e. p<0.001) the overall relative risk will be considered the most reliable guide to the approximate treatment effect in all patients.

**(c) Age**

There is evidence that fibrinolytic activation following TBI is greater in older patients ^26^. For this reason, we will examine the effect of tranexamic acid on head injury death stratified by age. We will examine three age strata: young (<30 years), middle (31–60 years), older (>60 years). Because time to treatment, TBI severity and SBP could confound the effect of age on treatment effectiveness, we will control for these variables. We will use interaction tests to see whether the effect of the treatment (if any) differs across these subgroups. We do not expect the effect of tranexamic acid to vary substantially by age and unless there is strong evidence against the null hypothesis of homogeneity of effects (i.e. p<0.001) the overall relative risk will be considered the most reliable guide to the approximate treatment effect in all patients.

### Secondary outcomes

*Early head injury death:* Because early head injury deaths are more likely to be the result of intracranial haemorrhage (since bleeding occurs early) than late head injury deaths (these are more likely due to non-bleeding causes that are unaffected by TXA), we will examine the effect of TXA on head injury deaths within 48 hours and 7 days of randomisation.

*Cause specific mortality:* We will assess and report the effect of tranexamic acid on all-cause mortality and cause specific mortality using relative risks and 95% confidence intervals. We will also present the distribution of causes of death by days since randomisation.

*Disability*: We assess disability using the Disability Rating Scale and with a set of disability questions based on discussions with victim representatives. The Disability Rating Scale measures the level of disability in six diagnostic categories: (1) eye opening, (2) best verbal response, (3) best motor response, (4) self-care ability for feeding, grooming and toileting, (5) level of cognitive functioning, and (6) employability. We can use the Disability Rating Scale across the span of recovery. The maximum score a patient can obtain is 29, which represents an extreme vegetative state. A person without disability would score zero. We will assess the effect of tranexamic acid on disability by comparing the mean Disability Rating Scale score in the tranexamic acid and placebo groups using parametric and non-parametric tests. We will report the effect of tranexamic acid on the patient derived disability measure by estimating the risk ratio of being in the extreme categories for each of the six areas of functioning (1. walking, 2. washing, 3. pain and discomfort, 4. anxiety or depression, 5. agitation or aggression and 6. fatigue).

*Vascular occlusive events:* By inhibiting fibrinolysis, tranexamic acid could increase the risk of cerebral ischaemia and thrombosis. Cerebral ischaemia is a common pathophysiological mechanism after TBI that can worsen neurological outcome and increase mortality. Raised intracranial pressure could lead to cerebral hypo-perfusion and thrombotic DIC might increase the risk of the cerebral micro-thrombi. These thrombi are often found in autopsies of patients with severe TBI. We will assess and report the effect of tranexamic acid on the risk ratio for fatal and non-fatal stroke both overall and stratified by time to treatment (less than versus more than 3 hours). The effect of tranexamic acid on cerebral infarction will be further evaluated using routine brain imaging from a selection of trial patients (sub-study to be published separately). The effect of tranexamic acid on other vascular occlusive events (fatal and non-fatal myocardial infarction, deep vein thrombosis and pulmonary embolism) will be reported both separately and combined.

*Seizures:* Tranexamic acid crosses the blood-brain barrier and in high dosages causes seizures. Although there was no increase in seizures in the CRASH-2 trial of tranexamic acid in extra-cranial bleeding, seizure activity is more common after TBI and remains a concern. We will therefore report the effect of tranexamic acid on the risk ratio for seizures.

*Neurosurgical interventions for intracranial haemorrhage:* If tranexamic acid treatment reduces intracranial bleeding it might reduce neurosurgical intervention for bleeding. On the other hand, if neurosurgical intervention precedes or coincides with administration of the trial treatment, there will be no opportunity for tranexamic acid to have an effect. We expect that including such patients in the analysis will dilute the treatment effect towards the null. Although tranexamic acid might not have enough time to reduce the need for neurosurgical intervention in the first place, it should have enough time to reduce the amount of bleeding during the operation. We will report the effect of tranexamic acid on haematoma evacuation and the mean blood loss in tranexamic acid and placebo treated patients. We will test the effect of treatment allocation on haematoma evacuation using relative risks and 95% confidence intervals. We will test the effect of treatment allocation on mean blood loss using a two-sample t-test.

*Complications:* Patients with TBI are at risk of other significant medical events including renal failure, sepsis and gastrointestinal bleeding and these outcomes are collected routinely.

*Adverse events:* We collect other untoward medical events up to 42 days after randomisation as adverse events (AEs). In line with ICH-GCP guidelines, an AE is considered as serious if it results in death, is life-threatening, requires inpatient hospitalisation or prolongation of existing hospitalisation, results in persistent or significant disability/incapacity, or in a congenital anomaly/birth defect ^16^. If there is a possibility that an AE is due to the trial drug, it is an adverse reaction. A suspected unexpected serious adverse reaction (SUSAR) is an unexpected occurrence of a serious adverse reaction. There need only be an index of suspicion that the event is a previously unreported reaction to a trial drug or a previously reported but exaggerated or unexpectedly frequent adverse drug reaction.

The number of AEs, SAEs, SUSARs grouped by MedDRA® codes and the number of patients with at least one event will be compared between arms using a chi-squared test (or Fisher’s exact test), with relative risks and 95% confidence intervals when these are computable.

## Other analyses: to be reported in separate publications

### Analysis 1. Reducing the impact of null bias

We will examine the timing of the effect of tranexamic acid on outcomes among patients treated within three hours of injury, by conducting repeated analyses excluding outcomes at increasing time intervals from randomisation. We will increase the length of the exclusion period by one hour at a time. This means that patients who die quickly following randomisation (who are more likely to have un-survivable injuries at baseline) can be excluded and the treatment effect evaluated without this null bias. Risk ratios and 95% confidence intervals will be estimated to assess the size and precision of the treatment effect.

### Analysis 2: Adjusting for possible imbalance in baseline prognostic factors

Given the size of the CRASH-3 trial, baseline characteristics that may influence the outcome should be evenly distributed between the treatment and placebo groups, so that any difference in outcome can be attributed to the intervention. However, it is still possible that a chance imbalance in important prognostic factors could influence the results. To investigate this possibility, we will conduct an analysis of the effect of treatment that is adjusted for baseline risk. We will build a prognostic model based on pre-specified baseline variables and use it to estimate the predicted risk of the outcome at baseline.

The primary outcome is head injury death. The most important prognostic factors for this outcome that are measured at baseline are age, GCS score, pupil reactivity and systolic blood pressure. These variables will be included in a multivariable prognostic model based on the final trial dataset. Although there are almost complete data on these variables, in the case of missing data, the missing values. The trial data will then be stratified into risk deciles as shown in the Table based on the predicted risk of the outcomes at baseline. We will report frequencies and percentages within each risk decile, and calculate a risk ratio (with 95% CI) for each risk decile. The pooled risk ratio (with a 95% CI) will be estimated as an inverse variance weighted average of the stratum specific risk ratios. The pooled risk ratio should provide an estimate of the treatment effect that is un-confounded by baseline risk. The advantage of this approach is that the effect of baseline risk on the treatment effect is more explicit than when covariate adjusted odds ratios are calculated using logistic regression. Furthermore, risk ratios are easier to interpret and apply to individual patients than are odds ratios.

A forest plot will be prepared to show graphically how the treatment effect varies by baseline risk. We will use a chi-squared test to assess any heterogeneity in treatment effect across the risk groups and we will calculate the I-squared statistic to quantify the percentage of variability in effect estimates that is due to heterogeneity rather than chance. To reduce the likelihood of making inappropriate inferences, we pre-specify that unless there is strong evidence against the null hypothesis of homogeneity of effects (i.e. P<0.001), the pooled relative risk will be considered the most reliable guide to the approximate treatment effects in all risk strata. We do not anticipate substantial heterogeneity by baseline risk.

### Analysis 3: Cost effectiveness

An economic analysis will be relevant if tranexamic acid clearly demonstrates efficacy in achieving its clinical aims. In this case, the study will be undertaken in the form of a cost-effectiveness analysis with the aim of estimating the incremental cost-effectiveness ratio comparing the use of tranexamic acid with normal clinical practice. Analysis will be based on adjusted life years gained. A further analysis will explore the use of the EQ-5D data to quality-adjusted survival. In this study, the economic analysis is clearly bounded as virtually all significant resource use will occur in the initial period of hospitalisation. As such, neither a long-term resource analysis nor an analysis of out of hospital costs will be required. The trial use of tranexamic acid is likely to mirror its use in normal clinical practice, hence the cost-effectiveness estimated in the trial (adjusted for protocol driven costs) will closely approximate cost-effectiveness in actual clinical practice. Data on physical resource consumption (e.g. length and nature of hospital stay) will be collected for each patient and a common unit cost at a country level will be applied. A sensitivity analysis will be undertaken to assess the robustness of the economic analysis in response to variations in key variables such as drug prices. In all cases, the economic analysis will be integrated with the clinical trial procedures to optimise efficiency and minimise inconvenience to patients. Time in the Intensive Care Unit (ICU) is the key resource consumption variable. Length of stay in the ICU and the hospital will be censored due to early deaths, or a stay in the ICU or hospital longer than 42 days. Summary statistics will include the median and the interquartile range computed separately for each treatment arm.

### Analysis 4: Examining the mechanism of action of tranexamic acid in TBI

An Intracranial Bleeding Mechanistic Sub-Study (IBMS) is nested in a cohort of approximately 1,000 CRASH-3 trial patients. This sub-study aims to examine the mechanism of action of tranexamic acid by evaluating brain images acquired before and after randomisation ^16^. Brain images are primarily examined for evidence of intracranial haemorrhage and cerebral infarction. Patients who have a GCS score of 12 or less or intracranial bleeding on a CT scan done before randomisation are eligible for inclusion. The results of the IBMS will be published when the CRASH-3 trial is complete. The IBMS protocol has been published and the associated statistical analysis plan will be published prior to completion of the CRASH-3 trial.

### Data monitoring and interim analyses

An independent Data Monitoring Committee is responsible for reviewing the progress of the CRASH-3 trial, including recruitment, data quality, and main outcomes and safety data. The DMC has the responsibility for deciding whether, while randomisation is in progress, the unblinded results (or the unblinded results for a particular subgroup), should be revealed to the Trial Steering Committee. They will do this if, and only if, two conditions are satisfied: (1) The results provide proof beyond reasonable doubt that treatment is on balance either definitely harmful or definitely favourable for all, or for a particular category of, participants in terms of the major outcome; and (2) The results, if revealed, would be expected to substantially change the prescribing patterns of clinicians who are already familiar with other trial results that exist. Exact criteria for “proof beyond reasonable doubt” are not, and cannot be, specified by a purely mathematical stopping rule, but they are strongly influenced by such rules. This is in agreement with the Peto-Haybittle stopping rule ^27^ whereby an interim analysis of major endpoint would generally need to involve a difference between treatment and control of at least three standard errors to justify premature disclosure. An interim subgroup analysis would have to be even more extreme to justify disclosure. This rule has the advantage that the exact number and timing of interim analyses need not be pre-specified. In summary, the stopping rules require extreme differences to justify premature disclosure and involve an appropriate combination of mathematical stopping rules and scientific judgment. To date, five interim analyses have been conducted by the Data Monitoring Committee with no recommendation for early stopping. These analyses were conducted in June 2012, March 2014, July 2015, May 2016 and December 2017, and involved the complete analysis of the un-blinded data as per the trial protocol. There was no change to the protocol as a result of the interim analyses and there are no more interim analyses planned. There are no interim analyses planned for the IBMS. The final analysis of the unblinded results will take place after recruitment is complete and the database is hardlocked.

### Data management and analysis software

All trial data is managed in accord with the CRASH-3 trial Data Management Plan (DMP) (version 1.1) and stored in the Trial Master File. The DMP standard operating procedures are produced in conjunction with the London School of Hygiene and Tropical Medicine (LSHTM) policies and procedures, the Clinical Trials Unit working procedures, and regulatory requirements. The clinical database management system for CRASH-3 trial and IBMS was built to comply with ICH-GCP. The database was developed by Sealed Envelope Ltd (UK). In the CRASH-3 trial, data are collected at each participating site and transmitted directly to the Clinical Trials Unit via the database. Where there is poor internet connection, the paper CRFs can be sent by fax or via email. Data checks and cleaning are performed by the Clinical Trials Unit. Data items to be coded including Adverse Event term and terms used to describe ‘other’ causes of death on the Outcome Form are coded using MedDRA Version 12. In the IBMS, the outcome data is directly uploaded onto an electronic database accessed at each sub-study site. The final database lock will take place at the end of the trial within three months from the time when the ‘Last patient’ in the ‘Last follow-up’ has completed the trial. Data will be exported for statistical analysis using the most recent version of Stata [StataCorp LP, College Station, Texas, USA].

### Dissemination of findings

The results of the CRASH-3 trial will be published in an established peer-reviewed journal. At least one publication of the main trial results will be made. Links to the publication will be provided in all applicable trial registers. Dissemination of results to patients will take place via the media, trial website and relevant patient organizations. In addition, participants and their families will be made aware of the trial results if requested. Collaborating investigators will play a vital role in disseminating the results to colleagues and patients. The success of the trial will be dependent entirely upon the collaboration of the nurses and doctors in the participating hospitals and those who hold key responsibility for the trial. Hence, the credit for the study will be assigned to the key collaborator(s) from each participating site, as it is crucial that those taking credit for the work have actually carried it out. The results of the trial will be reported first to trial collaborators. As a large number of hospitals in many countries will contribute to this trial, individual countries or sites cannot restrict the publication of the manuscript relating to the outcomes of this trial. Anonymous data for this trial will be made available for free use at The Free Bank of Injury and emergency Research Data (freeBIRD) website. Following publication of the primary and secondary analyses detailed in this statistical analysis plan, the trial data will be made available via our data sharing portal - The Free Bank of Injury and emergency Research Data (freeBIRD) website. This will allow for maximum utilization of the data to improve patient care and advance medical knowledge.

### Study status

The trial is currently recruiting patients and at the time of writing a total of 11,500 patients had been enrolled (target sample size 13,000).

## Discussion

This statistical analysis plan is an update to our previously published protocols. The main changes are: an increased sample size from 10,000 to 13,000 patients, and a change in the primary end point to death in hospital within 28 days of injury among patients randomised within 3 hours of injury but with a focus on very early treatment. We present our plan for the statistical analyses in advance of the database lock and un-blinding to guard against data dependent analyses. The CRASH-3 trial should provide reliable evidence on the effect of tranexamic acid on death and disability in patients with TBI.

## Data availability

No data are associated with this article.

## Supplementary material

Supplementary File 1: Trial entry form.

Supplementary File 2: CRASH-3 trial personnel and collaborators.
