# Supplementary material for: Tranexamic acid for significant traumatic brain injury (The CRASH-3 trial): Statistical analysis plan for an international, randomised, double-blind, placebo-controlled trial
Source: Wellcome Open Res. 2018 Sep 26;3:86. Originally published 2018 Jul 20. [Version 2] doi: 10.12688/wellcomeopenres.14700.2 (PMC6081978; doi:10.12688/wellcomeopenres.14700.2)
Supplement: Supplementary file 1 [file wellcomeopenres-3-16185-s0000.tgz › a4500eb1-7910-4ad1-ac1c-d7b551bfc547.pdf]

CRASH-3
Clinical Randomisation of an Antifibrinolytic in Significant Head Injury

ENTRY FORM

PLEASE COMPLETE 1-16 BEFORE RANDOMISING THE PATIENT

ABOUT YOUR HOSPITAL *(please ensure all information below is contained in the medical records)*

**1.** Country	
**2.** Hospital code *(in your Study File)*	

ABOUT THE PATIENT

**3.** Patient's initials *(first name/last name)*			**4.** Patient hospital ID		
**5.** Age *(years – approximate if unknown)*			**6.** Sex *(circle)*	**MALE**	**FEMALE**

ABOUT THE INJURY AND PATIENT'S CONDITION

**7.**	Time since injury *(insert hours)*		*Best estimate from history*
**8.**	Systolic Blood Pressure		*mmHg (most recent measurement prior to randomisation)*

9. Glasgow Coma Score (GCS) *(circle one response for each category)*

First measurement in hospital of GCS (if unknown give value at randomisation)

9A–EYE OPENING	9B–MOTOR RESPONSE	9C–VERBAL RESPONSE
4 Spontaneous	6 Obeys commands	5 Orientated
3 To sound	5 Localising	4 Confused speech
2 To pain	4 Normal flexion	3 Words
1 None	3 Abnormal flexion	2 Sounds
	2 Extending	1 None
	1 None	

IF GCS MORE THAN 12 AND NO CT SCAN AVAILABLE – __DO NOT RANDOMISE__

IF GCS MORE THAN 12, CT SCAN __IS__ AVAILABLE AND INTRACRANIAL BLEEDING=YES – __RANDOMISE__

**10.**	This GCS is *(circle one)*	**BEFORE**	**AFTER**	intubation/sedation

**11.**	Pupil reaction	**BOTH REACT**	**ONE REACTS**	**NONE REACT**	**UNABLE TO ASSESS**

**12.**	Any significant extracranial bleeding?	**YES**	**NO**	*Patients with extracranial trauma who are likely to need an early blood transfusion in the view of the attending doctor after taking into account mechanism of injury, findings from secondary survey, physiology and response to fluid infusion – __DO NOT RANDOMISE__*

**13.**	Any intracranial bleeding on CT scan (before randomisation)? *(circle one)*	**YES**	**NO**	**NO CT SCAN AVAILABLE**	*IF CT SCAN AVAILABLE AND INTRACRANIAL BLEEDING=NO – __DO NOT RANDOMISE__*

14. Location of intracranial haemorrhage on CT Scan *(circle one response for each line)*

a) Epidural	**YES**	**NO**
b) Subdural	**YES**	**NO**
c) Subarachnoid	**YES**	**NO**
d) Parenchymal	**YES**	**NO**
e) Intraventricular	**YES**	**NO**

RANDOMISATION INFORMATION

Eligible if adult, with TBI, no significant extracranial bleeding, within 8h of injury (GCS=12 or less, or any intracranial haemorrhage on CT scan)

**15.** Eligible? *(circle)*	**YES**	*Get the lowest available number treatment pack and follow instructions*	**NO**	*Do not randomise, record on screening log*

**16.** Consent process for entry used? *(circle)*	**WAIVER**	**OTHER REPRESENTATIVE**	**RELATIVE**

**17.** Insert treatment pack number here	**BOX**					**PACK**		

**18.** Date of randomisation	*day*	*month*	*year*	**19.** Time of randomisation *(24-hour clock)*	*hours*	*minutes*
**20.** Name of person randomising				**21.** Signature		

SEE GUIDANCE OVERLEAF

DATA FORMS GUIDANCE

AFTER COMPLETING THIS PAPER FORM PLEASE SEND THE DATA BY ANY METHOD LISTED:

- Enter these data directly into the trial database (username and password required)
- Upload as a secure scanned document (see Study File for details)
- Fax to +44 20 7299 4663

PLEASE STORE THE ORIGINAL FORM IN THE INVESTIGATOR'S STUDY FILE

PLEASE GIVE A COPY OF THIS COMPLETED FORM TO THE PERSON RESPONSIBLE FOR COMPLETING THE OUTCOME FORM AT YOUR HOSPITAL.

FOR UNBLINDING, ADVICE ON SERIOUS ADVERSE EVENT REPORTING AND OTHER URGENT ENQUIRIES PLEASE TELEPHONE +44(0)7768 707500
