# Supplementary material for: Tranexamic acid for significant traumatic brain injury (The CRASH-3 trial): Statistical analysis plan for an international, randomised, double-blind, placebo-controlled trial
Source: Wellcome Open Res. 2018 Sep 26;3:86. Originally published 2018 Jul 20. [Version 2] doi: 10.12688/wellcomeopenres.14700.2 (PMC6081978; doi:10.12688/wellcomeopenres.14700.2)
Supplement: Supplementary file 2 [file wellcomeopenres-3-16185-s0001.tgz › 5edb07c6-6864-4492-aeb8-3c56f7cfa3ff.docx]

**CRASH-3 Trial personnel and collaborators**

**Writing Committee:**

Ian Roberts (Chair), Tony Belli, Amy Brenner, Rizwana Chaudhri, Bukola Fawole, Tim Harris, Rashid Jooma, Abda Mahmood, Temitayo Shokunbi, Haleema Shakur.

**TSC** Peter Sandercock (Chair), Henry Benjamin Hartzenberg, Manjul Joshipura (2011-2016), Amy Aeron-Thomas, Ian Roberts, Pablo Perel, Haleema Shakur

**DMEC** Professor Samuel C Ohaegbulam, Professor Anthony Rodgers, Professor Mike Clarke

**Trial Co-ordinating Centre staff:** Emma Austin (assistant trial manager), Eni Balogun (trial manager), Lin Barneston (data manager), Danielle Beaumont (senior trial manager/research fellow), Imogen Brooks (trial assistant), Madeleine Cargill (data assistant), Lisa Cook (assistant trial manager), Beatrice Cornu-Hewitt (trial assistant), Phil Edwards (statistician), Lauren Frimley (acting trial manager), Amber Geer (data assistant), Daniel Gilbert (data assistant), Catherine Gilliam (trial administrator), Daniel Hetherington (trial assistant), Rob Jackson (data manager), David I’anson (assistant trial manager), Sneha Kansagra (assistant trial manager), Taemi Kawahara (senior trial manager), Katharine Ker (lecturer), Sergey Kostrov (systems officer), Hakim Miah (IT systems manager), Bernard Ndungu (assistant trial manager), Cecilia Okusi (data assistant), Aroudra Outtandy (trial assistant), Raul Pardinaz-Solis (assistant trial manager), Daniel Pearson (data assistant), Tracey Pepple (data manager), David Prieto-Merino (statistician), Danielle Prowse (assistant data manager), Nigel Quashi (data manager), Anna Quinn (data assistant), Maria Ramos (senior project administator), Mia Reid (clerical assistant), Ian Roberts (chief investigator/CTU co-director), Haleema Shakur (project director/ CTU co-director), Jemma Tanner (clinical trials associate), Andrew Thayne (data assistant), Lesley Vidaurre (assistant trial manager), Elizabeth Woods (assistant trial manager).

**Nigeria coordinating team:** Olusade Adetayo (assistant trial coordinator), Bukola Fawole (coordinating centre director), Olujide Okunade (assistant trial coordinator), Temitayo Shokunbi (clinical lead).

**Pakistan coordinating team:** Rizwana Chaudhri (coordinating centre director), Kiran Javaid (assistant research coordinator), Rashid Jooma (clinical lead), Aasia Kayani (research coordinator).

**CRASH-3 trial collaborators:**

*Afghanistan –* Nangarhar University Teaching Hospital, Abdul Basir Mangal

*Albania* – University Hospital of Trauma, Fatos Olldashi

*Cambodia* – World Mate Emergency Hospital, Simon Stock

*Cameroon* - Hopital General Douala, Mireille Moumi; Hopital Laquintinie de Douala, Joseph Fondop; Yaounde Central Hospital, Vincent Djientcheu; Yaounde General Hospital, Mba Sebastien

*Canada* – Saint John Regional Hospital, James French

*Colombia* – Fundacion Valle del Lili, Jorge H Mejia-Mantilla; Hospital Pablo Tobon Uribe, Alfredo Constain Franco; Hospital Universitario San Vicente Fundacion, Carlos Morales Uribe

*Egypt* – Mataria Teaching Hospital, Hussein Khamis

*El Salvador* – Hospital Nacional Rosales, Juan Tobar

*Georgia* – Archangel St Michael Multiprofile Clinical Hospital, Buba Shalamberidze; City Hospital 1, Gia Tomadze; High Technology Medical Center, University Clinic, Tamar Gogichaishvili

*Indonesia* – Rumah Sakit Sekar Kamulyan, Antonius Kurniawan

*Iraq* – Par Private Hospital, Dr Qadamkhear Hama; Rozhawa Emergency Hospital, Dr Qadamkhear Hama

*Ireland* – Cork University Hospital, Conor Deasy

*Italy* – Azienda Ospedaliera Universitaria Senese, Paola Bonucci; Fondazione Poliambulanza, Alan Girardini; Spedali Civili Di Brescia, Frank Rasulo

*Jamaica* - Cornwall Regional Hospital, Jeffrey East; University Hospital of the West Indies, Jean Williams-Johnson

*Japan* – Matsudo City Hospital, Tomohisa Shoko; Senshu Trauma and Critical Care Centre (STCCC), Yasushi Hagihara; Teikyo University Hospital, Takahiro Ohnuki; Tokyo Medical and Dental University, Yasuhiro Otomo

*Malaysia* – Hospital Kemaman, Mohd Zaki Fadzil bin Senek; Hospital Kulim, Shamsul Anuar Bin Asmee; Hospital Langkawi, Munirah Osman; Hospital Raja Permaisuri Bainun, Adi Bin Osman; Hospital Seberang Jaya, Noor Azleen Bt Ayop; Hospital Segamat, Ahmad Abdul Basitz Bin Ahmad Fatan; Hospital Sultan Abdul Halim, Zainal Effendy Zainal Abidin; Hospital Sultanah Aminah, Mohd Amin Bin Mohidin; Hospital Sultanah Bahiyah, Fatahul Laham B Mohamed; Hospital Sultanah Nur Zahirah, Hamzah Mohd Lotfi; Hospital Sungai Buloh, Sabariah Faizah Bt Jamaluddin; Hospital Tengku Ampuan Afzan, Zainalabidin Bin Mohamed Ismail; Hospital Tuanku Fauziah, Ida Zaliza Zainol Abidin; Miri General Hospital, Chia Boon Yang; Penang General Hospital (Pulau Pinang), Kwanhathai Darin Wong; Universiti Sains Malaysia, Kamarul Aryffin Baharuddin

*Mexico -* Hospital General Jose G Parres, Nancy Guevara Rubio; Hospital General La Perla, Angelica Soria Huerta; Hospital General Regional Bernardo Sepulveda, Hafid Eliacim Sanchez Flores; Hospital General Regional No.1, Francisco Javier Estrada Arreola; Hospital General de Uruapan, Dr Pedro Daniel Martinez, Patricia Ortega Leon; Hospital General de Zona 197, Erandy Montes de Oca Garcia; Hospital Regional 25 IMSS, Jorge Loria Castellanos

*Myanmar*- 1000 Bedded Nay Pyi Daw Hospital, Than Latt Aung

*Nepal* – B & B Hospital (P) Ltd., Krishna Sharma; Bir Hospital, Rajiv Kumar Jha; Chitwan Medical College Teaching Hospital, Ajit Shrestha; Kathmandu Medical College Teaching Hospital, Amit Thapa; National Institute of Neurological and Allied Sciences (NINAS), Upendra Devkota; Neuro Hospital, Yam Bahadur Roka

*Nigeria* – Abubakar Tafawa Balewa University Teaching Hospital, Azeez Tella; Bowen University Teaching Hospital (BUTH) Ogbomoso, Adewumi Durodola; Federal Medical Centre Abeokuta, Fidelis E Ojeblenu; Federal Medical Centre Bida, John James Adeniran; Federal Medical Centre Ido-Ekiti, Olakunle Fatai Babalola; Federal Medical Centre Lokoja, Ukpong Udoffa; Federal Medical Centre Umuahia, Uchechi Ekpemiro; Federal Teaching Hospital, Gombe, Dr Mohammed Ibrahim Guduf; Irrua Specialist Teaching Hospital, Andrew Ezivbashe Dongo; Jos University Teaching Hospital, Danaan Shilong; Lagos State Accident and Emergency Centre, Ajibola Fatai Ishola; Lagos University Teaching Hospital, Femi Bankole; National Hospital Abuja, Olaomi Oluwole; Nnamdi Azikiwe University Teaching Hospital, Stanley Anyanwu; Obafemi Awolowo University Teaching Hospitals, Edward Komolafe; Olabisi Onabanjo University Teaching Hospital, Lateef OA Thanni; State Hospital, Ijaiye, Abeokuta, Nafiu Aigoro; University College Hospital, Ibadan, Adefolarin Malomo; University of Abuja Teaching Hospital, Joseph Orinya Obande; University of Benin Teaching Hospital, Dr Abiodun Azeez; University of Nigeria Teaching Hospital Enugu, Wilfred Mezue

*Pakistan* – Aga Khan University Hospital, Rashid Jooma; DHQ Khuzdar, Abdul Wahid; DHQ Narowal, Muhammad Sajjad Yaqoob; DHQ Rawalpindi, Nadeem Akhtar; Jinnah Hospital Lahore, Naveed Ashraf; Jinnah Postgraduate Medical Centre (JPMC), Lal Rehman; Lady Reading Hospital, Mumtaz Ali; Lahore General Hospital Neurosurgery Unit I, Rizwan Masood Butt; Lahore General Hospital Neurosurgery Unit II, Khalid Mahmood; Lahore General Hospital Neurosurgery Unit III, Asif Bashir; Liaquat National Hospital and Medical College, Salman Yousuf Sharif; Liaquat University Hospital (LUMHS), Riaz Ahmed Raja Memon; Services Hospital Lahore, Samra Majeed; Shifa International Hospital, Abdus Salam Khan

*Papua New Guinea* - Port Moresby General Hospital, Bobby Wellsh

*Romania* – Bagdasar-Arseni Emergency Clinical Hospital, Mircea Radu Gorgan; Spitalul Sf. Pantelimon Bucharest, Adam Danil; Timisoara County Hospital, Horia Ples

*Slovenia* - University Medical Centre Ljubljana, Dušan Vlahović

*Spain* – Complejo Hospitalario de Navarra, Juan Angel Tihista Jimenez; Hospital Alvaro Cunqueiro (formerly Hospital Xeral-Cies) VIGO, Melida Garcia Martul; Hospital Clinico de Barcelona, Jaime Fontanals Dotras; Hospital de Lucus Augusti, Laura Rodriguez Peralta; Hospital General Universitario De Albacete (CHUA), Joaquin F Paya Berbegal; Hospital General Universitario de Ciudad Real, Maria Carmen Corcobado Marquez; Hospital Regional Universitario Carlos Haya, Juan F Fernandez-Ortega; Hospital Torrecardenas, Susana Maria Parra Alonso; Hospital Universitario Puerta del Mar de Cadiz, Jose Manuel Jimenez Moragas; Hospital Universitario Virgen del Rocio, Claudio Garcia Alfaro

*United Arab Emirates* - Al Qassimi Hospital, Satish Krishnan

*United Kingdom* – Addenbrooke's Hospital, Adrian Boyle; Aintree University Hospital, Abdo H Sattout; Arrowe Park Hospital, Andrea Wootten; Basingstoke and North Hampshire Hospital, Louisa Chan; Birmingham Heartlands Hospital, Susan Elizabeth Dorrian; Blackpool Victoria Hospital, Simon Tucker; Darlington Memorial Hospital, Ola Afolabi; Derriford Hospital, Anthony Desmond Kehoe; Dorset County Hospital, Tamsin Ribbons; Glasgow Royal Infirmary, Alastair Ireland; Gloucestershire Royal Hospital, Tanya De Weymarn; Great Western Hospital, Stephen Haig; Gwynedd Hospital, Bangor, Rob Perry; Hull Royal Infirmary, William Townend; James Cook University Hospital, Judith Wright; John Radcliffe Hospital, Melanie C Darwent; King's College Hospital, Philip Anthony Hopkins; Leeds General Infirmary, Tajek Basheer Hassan; Leicester Royal Infirmary, Timothy John Coats; Manchester Royal Infirmary, Richard Body; Milton Keynes University Hospital, Shindo Puthoor Francis; Monklands Hospital, Fiona Hunter; Musgrove Park Hospital, James Gagg; Newham University Hospital, Tim Harris; Norfolk and Norwich University Hospital, Meenal Galal; North Devon District Hospital, Liam Kevern; Northern General Hospital, Avril Kuhrt; Pinderfields General Hospital, Sarah Robertshaw; Poole Hospital, Henrick Reschreiter; Queen Alexandra Hospital, Christiane Vorwerk; Queen Elizabeth Hospital Birmingham, Antonio Belli; Queen Elizabeth University Hospital, Cieran McKiernan; Queen's Medical Centre, Frank Coffey; Royal Alexandra Hospital, Alasdair Corfield; Royal Berkshire Hospital, Liza Keating; Royal Cornwall Hospital, Mark Antony Ravi Jadav; Royal Liverpool Hospital, Jane McVicar; Royal London Hospital, Ben Bloom; Royal Oldham Hospital, Gabrielle May; Royal Preston Hospital, Andy Curran; Royal Stoke University Hospital, Julie Norton; Royal Sussex County Hospital, Rowley Cottingham; Royal United Hospitals Bath, David Watson; Salford Royal Hospital, Fiona Lecky; Southampton General Hospital, Diederik Olivier Bulters; Southmead Hospital, Jason Kendall; St George's Hospital, Phil Moss; St Mary's Hospital, London, Mark Wilson; University Hospital Coventry, Caroline Leech; University Hospital Lewisham, Hyun Choi; University Hospital of North Tees, Kayode Adeboye; Western Infirmary, Claire McGroarty; Wexham Park Hospital, Sarah Wilson; Whipps Cross University Hospital, Tim Haris; Whiston Hospital, Himanshu Kataria; Whittington Hospital, Lucy Parker; Worcestershire Royal Hospital, Paul Mathias Arthur Shone; Worthing Hospital, Mandy Grocutt; Yeovil District Hospital, Katalin Fernando

*Zambia* - Kitwe Central Hospital, Jonathan Mulenga; University Teaching Hospital Lusaka, Yakub Mulla.
