# Supplementary material for: Tranexamic acid for significant traumatic brain injury (The CRASH-3 trial): Statistical analysis plan for an international, randomised, double-blind, placebo-controlled trial
Source: Wellcome Open Res. 2018 Sep 26;3:86. Originally published 2018 Jul 20. [Version 2] doi: 10.12688/wellcomeopenres.14700.2 (PMC6081978; doi:10.12688/wellcomeopenres.14700.2)
Supplement: Peer review history

# Peer review: Referee response for version 1

**DOI:** 10.21956/wellcomeopenres.16009.r33536

**Published:** 2018-09-21

**Authors:** Susanne May

**Affiliations:** 1 The Clinical Trials Center, Department of Biostatistics, University of Washington, Seattle, WA, USA

Comments: (page numbers refer to the pdf version)

 Abstract

 One of the major reasons for the change is the focus on patients receiving treatment within 3 hours of injury and the sample size was modified to accommodate this. It is unclear from the abstract though what the (new) target enrolment number is for those patients that receive treatment within 3 hours. This also does not seem to be clear from the text. There should either be a mentioning of the revised target sample size for those who receive treatment within three hours or a justification why that is not given.

 It would be helpful to state in the abstract the timeframe for the mortality outcomes for the secondary outcomes (are they the same as for the primary?).

 It is unclear (but might difficult to explain concisely) what is meant by subgroup .. stratified by “baseline risk”. How is baseline risk defined?

 Generally

 In the emergency setting it is difficult to imagine that the time from injury to treatment can be measured exactly. It would be helpful to discuss this at least to some degree and to consider adding the word “estimated” if that is judged to be appropriate.

 Another subject that would be good to provide a little further information (unless it is in one of the referenced docs) is how “discharged alive” is defined. E.g. what about discharge to hospice?

 “list of abbreviations section” SUSARs: Suspected unexpected rather than Suspected unsuspected.

 Regarding page 3 left column at the bottom: would be good to mention “blinded” here as well.

 Regarding section Randomisation and masking: might be helpful to mention the TXA dose here as well.

 Regarding section “sample size”, I don’t understand why the updated sample size calculations can not be provided. If there is a reason for why, the reason should be given/described.

 Regarding section “Baseline characteristics” At the end of the paragraph it is indicated that baseline characteristics of each group will be described with frequencies and percentages. Is that sufficient? How about for continuous variables? Or are they all dichotomized or divided into categories?

 Regarding page 5 left column second paragraph, “however…” it seems that if this is the primary justification, the article was published in 2011, why wait until 2016 to make the change? Just an additional comment regarding the reason for the time lapse would be good.

 Regarding Primary analysis: If I am not mistaken, two measures (relative and absolute risks) are suggested for the primary analysis. This can lead to multiple comparison issues and what would be done if the results were contradictory? It would be best to choose one specific analysis for the primary analysis and suggest the other one as secondary.

 Regarding Missing data: Is there any evidence/data to suggest that patients (or family members) might withdraw consent?

 Regarding Figure 1: Did I miss it, or what were the data the graphs are based on? Where do these data for the graphs come from?

 Regarding secondary outcomes: … “will examine the effect of TXA on head injury deaths within 48 hours and 7 days of randomisation”. This could be problematic, if TXA were to contribute to more deaths within the first 48 hours.

 Regarding section “disability”: at what time point(s) are those disability measures taken. Why are both parametric and non-parametric tests proposed? What would be the conclusions if the test disagree? How would adjustments be made for multiple comparisons if any?

 Regarding section: “Neurosurgical intervention for intracranial haemorrhage” why aren’t specific statistical test proposed in other sections except here (t-test)?

 Regarding “Other analysis: to be reported in separate publications” Excluding early deaths is said to allow evaluation of treatment effect “without this null bias”. But if TXA has some effect on early patient survival, the result might be that patients on TXA are sicker if early deaths are excluded.

 Regarding p8 left column paragraph starting with “the primary outcome…”: “missing values will be replaced by the mean of the observed data” such single imputation approach will lead to underestimating the variability in the data and depending on the actual amount of missingness (even though the authors assume it is going to be a small amount) can lead to serious bias in estimating the variability in the data and should be avoided.

 Regarding p8 right column: “However, we hypothesise that the risk reduction with tranexamic acid would be greatest in patients at low risk of death since a smaller proportion of these patients will have un-survivable TBI”. This depends on how “low risk” is defined, as in this population one might expect a certain percentage of patients who get better without even needing TXA (thus at low risk, e.g. patients who are intoxicated and have a TBI, it is very difficult to tell at the time of treatment initiation how severely they are actually injured and whether or not they would need any TXA).

 Regarding Analysis 3: censoring deaths for length of ICU stay: similar to excluding early deaths, censoring of deaths for this analysis could seriously bias the results as “censoring” in the analysis typically carries the assumption of “independent censoring” which is unlikely to be true. Also, if TXA were to have any beneficial impact on early survival this might result in a longer ICU stay on average for this group and would be problematic in this analysis.

 Regarding line 4 left column p9: write out abbreviation TSC and line -13 write out abbreviation PHP.

I have read this submission. I believe that I have an appropriate level of expertise to confirm that it is of an acceptable scientific standard, however I have significant reservations, as outlined above.

# Peer review: Referee response for version 1

**DOI:** 10.21956/wellcomeopenres.16009.r33535

**Published:** 2018-08-20

**Authors:** Deepak Kumar Gupta, Anna Teresa Mazzeo

**Affiliations:** 1 Department of Anaesthesia and Intensive Care, Università di Torino, Torino, 10126, Italy; 2 Department of Neurosurgery, Neurosciences Centre, All India Institute of Medical Sciences, Delh (AIIMS), New Delhi, Delhi, India

Early administration of TXA ( tranexamic acid) within 3 hrs safely reduced the risk of death in bleeding trauma patients and was found to be highly cost-effective in CRASH 2 study of 20211 patients [ which included polytrauma subjects with traumatic brain injury also] ^1^. CRASH-3 study aims to evaluate the effectiveness and safety of TXA in the treatments of isolated brain injury patients. It is believed that early administration of tranexamic acid in TBI patients might limit intracranial bleeding, reducing death and disability. 

 Present study [CRASH-3] now plans to recruit 13000 TBI patients [ Sample size increased by 3000  to get enough patients within 3 hours of injury instead of earlier proposed 8 hrs ] for randomisation to tranexamic acid or matching placebo in addition to usual care. The primary outcome is head injury death in hospital within 28 days of injury for patients treated within 3 hours of injury.  Recruitment is unlikely to be an issue [due to recent  protocol update to include patients within 3 hours of injury] as 11500 patients have already been included in the study [hoping that all/majority of them were randomised and treated within 3 hours of injury]. Null hypothesis of homogeneity is not required in this study due to available evidence from CRASH 2 study on the same drug.

 Worldwide, about 50 million people have a TBI each year, and it is estimated that about half the world’s population will have one or more TBIs over their lifetime. Approximately 50% of trauma deaths are likely related to TBI which would imply that about one TBI-related death occurs every 3 minutes in some nations (India). Many epidemiological studies based on comparative effectiveness research model (CER) are currently underway/completed recruitments to better characterize TBI and to identify effective clinical interventions for TBI care. Centre TBI for adults and ADAPT for children have completed recruitments of over 5000 ( Adults) and 1000 ( children) already and their results are likely to throw further light on our understanding of traumatic brain injuries ^2^.

 In- hospital  28 days mortality end point analysis as study drug effect may not translate to long term outcome in TBI subjects for various reasons.We documented influence of achieving early ICU adherence to  head trauma guidelines with significantly lower in-hospital mortality in comparative study of two centres. Although long-term outcomes generally improved, patients discharged with favourable GOS score often deteriorated at home thereby implying influence of need of rehabilitation care for TBI subjects [ CHIRAG Study] ^3^.

 TBI population is quite heterogenous both in terms of mechanism of injury and in prehospital /in-hospital treatment delays.  It may be noted that in some of developing nations prehospital services are still lacking for most of TBI subjects. Loss of follow up data margin of 1% sounds a bit too optimistic as some of TBI subjects who live in far off catchments areas may not be available for follow ups after initial discharge from hospital. 

 Current statistical analysis plan with increased patient number within three hours of injury and  primary end point of death in hospital within 28 days of injury amongst randomized looks fine and is likely to provide evidence on effect of this drug on death and disability in patients with TBI. However, for developing nations where prehospital infrastructure is still lacking, it will be worthwhile looking at results of this drug or patients treated within 3-8 hours of injury too.

We have read this submission. We believe that we have an appropriate level of expertise to confirm that it is of an acceptable scientific standard.

## References

[1] CRASH-2 trial collaboratorsShakurHRobertsIBautistaRCaballeroJCoatsTDewanYEl-SayedHGogichaishviliTGuptaSHerreraJHuntBIribhogbePIzurietaMKhamisHKomolafeEMarreroMAMejía-MantillaJMirandaJMoralesCOlaomiOOlldashiFPerelPPetoRRamanaPVRaviRRYutthakasemsuntS: Effects of tranexamic acid on death, vascular occlusive events, and blood transfusion in trauma patients with significant haemorrhage (CRASH-2): a randomised, placebo-controlled trial. *Lancet*.2010;376(9734) : 10.1016/S0140-6736(10)60835-5 23-32 10.1016/S0140-6736(10)60835-5 20554319

[2] MaasAIRMenonDKAdelsonPDAndelicNBellMJBelliABraggePBrazinovaABükiAChesnutRMCiterioGCoburnMCooperDJCrowderATCzeiterECzosnykaMDiaz-ArrastiaRDreierJPDuhaimeACErcoleAvan EssenTAFeiginVLGaoGGiacinoJGonzalez-LaraLEGruenRLGuptaDHartingsJAHillSJiangJYKetharanathanNKompanjeEJOLanyonLLaureysSLeckyFLevinHLingsmaHFMaegeleMMajdanMManleyGMarstellerJMasciaLMcFadyenCMondelloSNewcombeVPalotieAParizelPMPeulWPiercyJPolinderSPuybassetLRasmussenTERossaintRSmielewskiPSöderbergJStanworthSJSteinMBvon SteinbüchelNStewartWSteyerbergEWStocchettiNSynnotATe AoBTenovuoOTheadomATibboelDVidettaWWangKKWWilliamsWHWilsonLYaffeKInTBIR Participants and Investigators: Traumatic brain injury: integrated approaches to improve prevention, clinical care, and research. *Lancet Neurol*.2017;16(12) : 10.1016/S1474-4422(17)30371-X 987-1048 10.1016/S1474-4422(17)30371-X 29122524

[3] GuptaDSharmaDKannanNPrapruetthamSMockCWangJQiuQPandeyRMMahapatraADashHHHeckerJGRivaraFPRowhani-RahbarAVavilalaMS: Guideline Adherence and Outcomes in Severe Adult Traumatic Brain Injury for the CHIRAG (Collaborative Head Injury and Guidelines) Study. *World Neurosurg*.89: 10.1016/j.wneu.2015.12.097 169-79 10.1016/j.wneu.2015.12.097 26806065PMC4870118

# Peer review: Referee response for version 1

**DOI:** 10.21956/wellcomeopenres.16009.r33537

**Published:** 2018-08-07

**Authors:** Shahriar Zehtabchi

**Affiliations:** 1 Department of Emergency Medicine, State University of New York, Downstate Medical Center, Brooklyn, NY, USA

This manuscript outlines the detail of the revised statistical analysis plan for CRASH-3 trial. The trial is being conducted to assess the impact of administration of tranexamic acid on the survival of patients with traumatic brain injury. The study design has been adjusted to improve the likelihood of identifying the benefit if it exists. This adjustment includes enrolling within a narrower window after head injury. This adjustment is justified by studies that proved the most benefit from TXA within the first 3 hours of brain injury. The investigators had to adjust  (increase) the sample size to maintain the study power at the pre-designed level.

 The details of the trial objectives, trial outcomes, and the methodology as well as the statistical analysis plan are well described. 

 Minor:

 - Page 5, under the section Primary Outcome, the authors state: "......The results showed that immediate treatment improved survival by more than 70% (OR 1.72, 95% CI 1.42-2.10)"  The number 70% is obviously the relative risk reduction. It would be more helpful for the readers to know the absolute risk reduction/difference. I was not able to calculate the absolute risk difference from the referenced original trial either.

 - Small trails published on this topic have measured the effect of TXA on the size of hematoma as well (in those with documented brain hematoma). This is clearly not a patient-centered outcome. However, it would have been interesting for the readers to know why the investigators do not wish to measure this outcome.

I have read this submission. I believe that I have an appropriate level of expertise to confirm that it is of an acceptable scientific standard.

# Peer review: Referee response for version 1

**DOI:** 10.21956/wellcomeopenres.16009.r33534

**Published:** 2018-07-25

**Authors:** Paul S. Myles

**Affiliations:** 1 Department of Anaesthesia and Perioperative Medicine, Alfred Hospital, Monash University, Melbourne, Vic, Australia

Excellent overview and justification, and highly relevant to eventual interpretation of the results and implementation. There are now consistent data showing a differential effect of TxA according to the timing of injury and administration.

 Although all-cause mortality is important and robust, disability-free survival (from the DRS) should if possible also be estimated.

 There are some data  suggesting that TxA may reduce infection risk, and so both mortality and other outcomes have pick this up (as opposed to it being "diluted").

I have read this submission. I believe that I have an appropriate level of expertise to confirm that it is of an acceptable scientific standard.
